# Addressing Flabby Ridges in Complete Denture Patients: A Case Report

**DOI:** 10.7759/cureus.64006

**Published:** 2024-07-07

**Authors:** Namita Zilpilwar, Sharayu Nimonkar, Surekha A Dubey, Vikram Belkhode

**Affiliations:** 1 Department of Prosthodontics, Sharad Pawar Dental College and Hospital, Datta Meghe Institute of Higher Education and Research, Wardha, IND

**Keywords:** hobkirk technique, prosthodontic, impression, flabby tissue, complete denture

## Abstract

A common clinical condition affecting the alveolar ridges of the mandibular or maxillary arches is known as a "flabby ridge." This condition involves a superficial region of movable soft tissue. It is particularly prevalent among long-term denture users, especially in the lower anterior region. Flabby ridges occur when hyperplastic soft tissue replaces the alveolar bone, resulting in mobile, hypermobile, and frequently edematous tissue. This condition is most commonly found in the edentulous areas of the oral cavity, especially in the maxillary anterior region. To provide a good fit for the patient, it is necessary to create a denture with accurate morphology, unique contours, and mobility of flabby tissue. The presence of a flabby ridge can significantly impact the stability, fit, and functionality of dentures. Management of flabby tissue includes various methods such as impression techniques, surgical intervention, and denture designing. This case report aims to provide an improved and controlled application of polyvinyl siloxane impression material in managing flabby tissue conditions, which is commonly used in dental practices. It presents a modified window technique for making impressions of anterior mandibular flabby tissues with their natural, undistorted state, leading to a more accurate and stable denture fit.

## Introduction

Hypermobile or flabby tissues in the denture-bearing region can make complete dentures problematic, causing pain when wearing the denture and leading to denture displacement. Restoring appearance, comfort, and functionality through the use of a stable prosthesis to replace lost teeth and alveolar tissues is the goal of complete denture prosthodontics [1]. For sufficient soft-tissue support for the denture, the masticatory mucosa's 1.5-2 mm thickness should ideally be laid on the residual ridge. According to studies, there are flabby ridges on about 5% of the edentate mandibles and 24% of the edentate maxillae [2].

The displacement or distortion of flabby tissues by force used during impression creation is the primary concern when a flabby ridge develops in the presence of anterior hyperfunction syndrome [3]. If dentures are made using this impression, they could not fit well. Flabby tissue can be managed by surgical intervention or by prosthetic intervention. Surgical intervention includes ridge augmentation, excision, and the injection of sclerosing solutions. Nevertheless, surgically excising the loose tissue results in an increase in the bulk of the denture material and the elimination of soft tissues that absorb stress, which damages the underlying tissues. Moreover, the treatment of dentures with flabby ridges more commonly involves the use of traditional prosthodontic treatments, including balancing occlusal loads and special impression techniques [4].

Again, there was a controversy over whether the static or compressive method was more suitable for the impression in the flabby tissue cases. Later, dentists supported the idea of using static techniques to record tissues. The mucostatic impression technique has been employed in areas where flabby tissue is found, utilizing the window tray technique, incorporating numerous relief holes, or employing double spacers. Because of mucosal alterations brought on by musculature dynamics or tissue irritation, as well as bone resorption, the dimensions of the edentulous ridges are unstable, as complete dentures rarely remain in contact with the surrounding mucosa [5].

A flexible denture foundation should be able to adjust to the mucosa continually. However, it must also support the teeth during function and, therefore, needs to be rigid. Naturally, a single material cannot have all of these qualities. Nevertheless, the foundation can be made of a variety of materials that allow it to be flexible when it comes into contact with soft tissues and hard where strength is required. Understanding the individual needs of each patient is crucial in formulating a comprehensive treatment plan. Incorporating tissue conditioning, modified impression techniques, and resilient denture liners can significantly improve denture stability and retention. This tailored approach ensures that the treatment aligns with the specific requirements of the patient, thereby enhancing the overall efficacy of the intervention [6].

This case report includes the use of the Zafarullah and Hobkirk technique, known as the modified window technique, for the management of the flabby ridge for prosthodontics rehabilitation. A valuable innovation described in this article is using polyvinyl siloxane (PVS) impression material instead of plaster of Paris in the case of flabby ridges in prosthodontic rehabilitation. The uniform application of plaster of Paris and controlling its low viscosity, especially when applied to flabby tissues, are a significant challenge. Patient positioning and the effect of gravity may further compound this problem, leading to inconsistent results and unreliable impressions [4].

The application of PVS impression materials definitely improves flabby ridge management as opposed to the use of plaster of Paris. This technique will eliminate the various disadvantages of plaster of Paris and, thus, provide prosthetic rehabilitation that is more accurate, stable, and reliable.

## Case presentation

**Figure 1:**
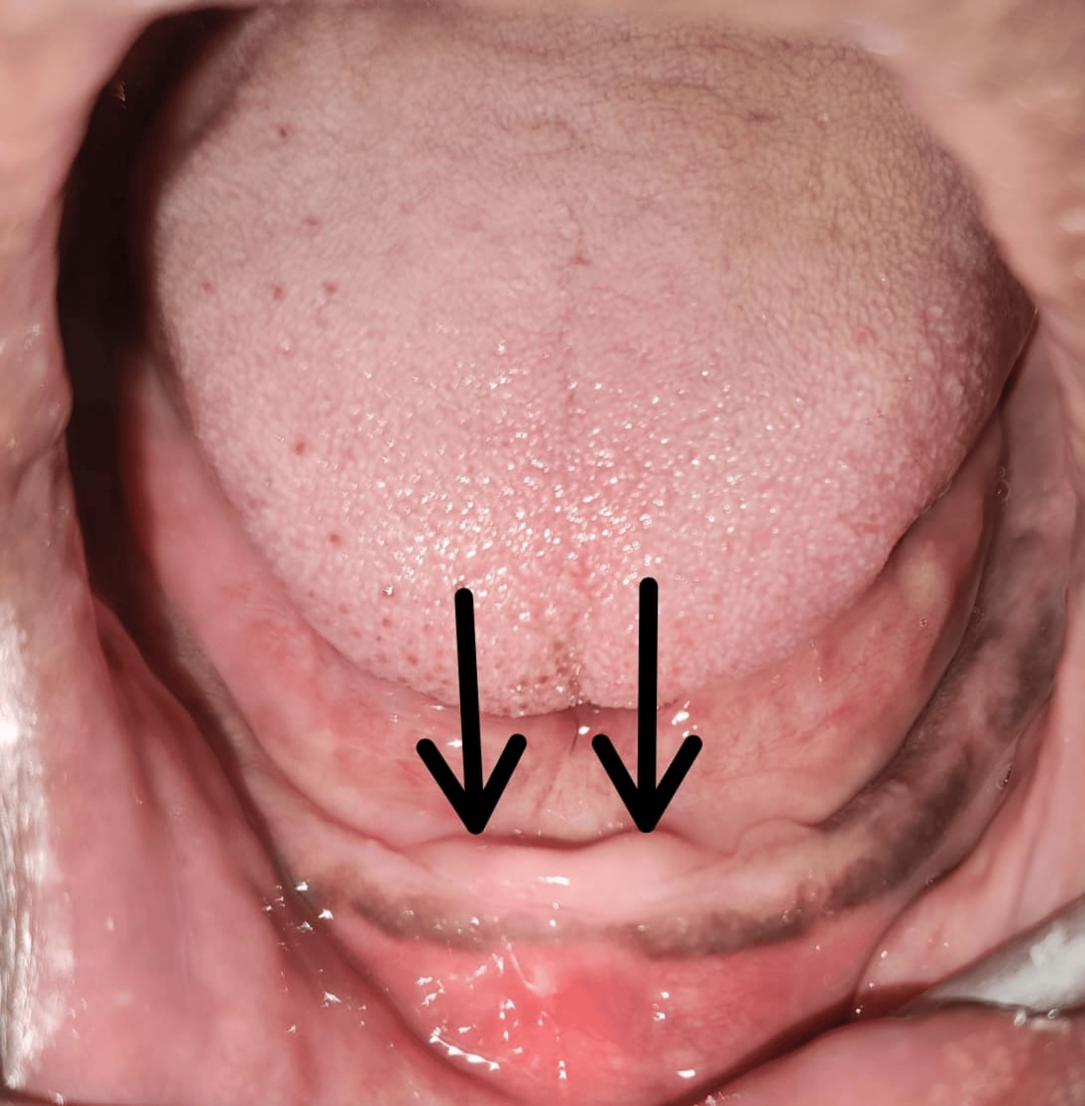
Flabby tissue seen in the mandibular anterior region of the jaw (black arrows)

A 56-year-old male patient reported to the Department of Prosthodontics, Crown, and Bridge with a chief complaint of missing teeth and wanted to get them replaced. After taking the patient's history, it was revealed that the patient is not a denture wearer but has a history of extraction in the lower anterior region of the jaw one year back. During the examination, flabby tissue was seen in the mandibular anterior region of the jaw after extraction of the alveolar bone resorbs, which resulted in substantial loss of bone over time. The ridge consisted mainly of fibrous tissue rather than compact bone, as shown in Figure 1. Complete denture rehabilitation was planned using a modified window technique. The patient was instructed to perform gentle gum massages daily to strengthen the tissues and promote blood circulation, which aids in tissue health and resilience. He was also advised to rinse his mouth with warm salt water several times daily. This practice helps reduce inflammation, promote healing, and maintain oral hygiene.

Impression procedure

**Figure 2:**
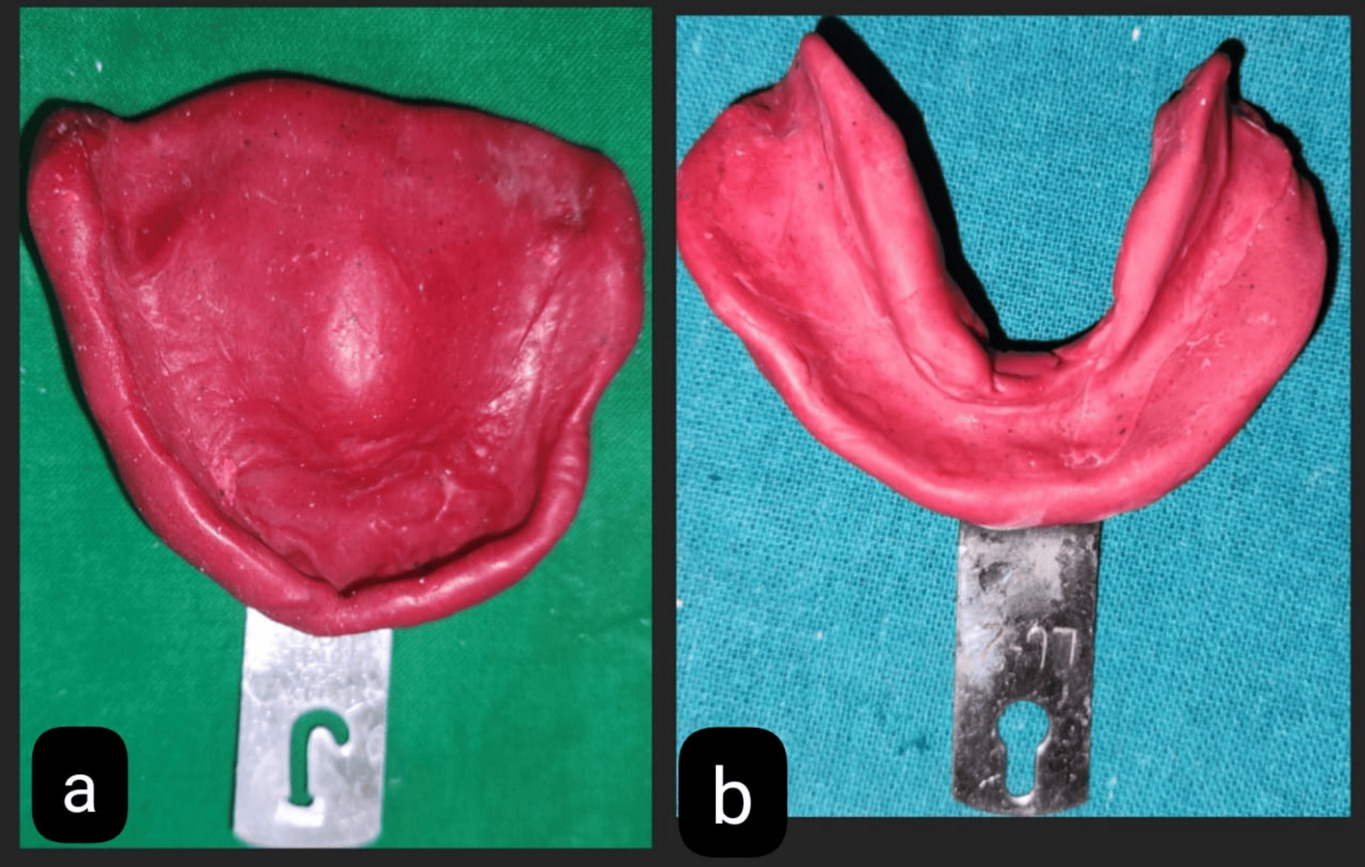
Preliminary impressions made with the type 2 impression compound of (a) maxilla and (b) mandible

Using impression compound (type 2), preliminary impressions were made at the first appointment for the mandibular and maxillary arches, as shown in Figures 2a, 2b. The primary cast was obtained by immediately pouring an impression into dental plaster (type II gypsum product) (Neelkanth Dentco, Jodhpur). For the final impression, the Zafarullah and Hobkirk technique was planned. In this technique, a set of procedures must be followed to precisely capture flabby tissue in its functional state.

*Custom Tray* *Fabrication*

A tray was made specifically to fit the patient's oral anatomy. Particular attention was paid to the areas of flabby tissue in the tray's construction.

Border Molding

A green stick thermoplastic impression compound (DPI Pinnacle, Mumbai) was used to shape the boundaries of the custom tray to capture the functional movements of the surrounding tissues. This phase ensured the flabby tissue was not moved by the tray's edges throughout the impression process.

Dual impression technique

Step 1

**Figure 3:**
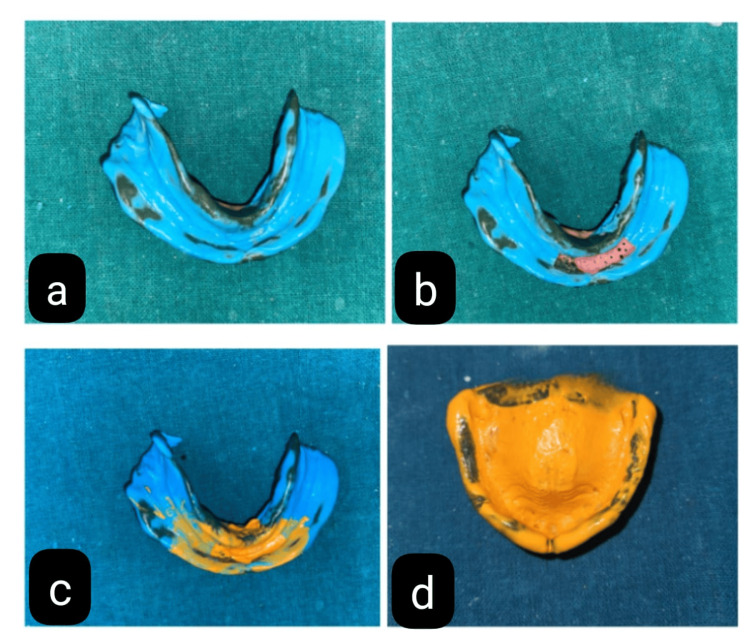
(a) Mandibular final impression with medium body elastomeric impression material. (b) Perforation in the flabby tissue region. (c) Final impression with light body elastomeric impression material. (d) Final impression of the maxillary arch

A custom tray of mandible was covered with medium body elastomeric impression material (Dentsply Reprosil Impression Material, Chattarpur) (Figure 3a) to make the final impression. After that, the tray was placed into the mouth, and the patient was told to make functional motions, including speaking, swallowing, and smiling. By using this procedure, the final impression precisely molded the location and shape of the flabby ridge without being compressed.

Step 2

The medium body impression material was scraped out from the region where flabby tissue was present, and the tray was perforated to make the impression stable (Figure 3b). Again, an impression with light body elastomeric impression material (Dentsply Reprosil Impression Material) in that region was made to record the anterior region in the static position of the tissue (Figure 3c). The impression is closely inspected to ensure that the flabby tissue has been faithfully captured in its original, uncompressed state. If necessary, the impression procedure is repeated to address any differences.

**Figure 4:**
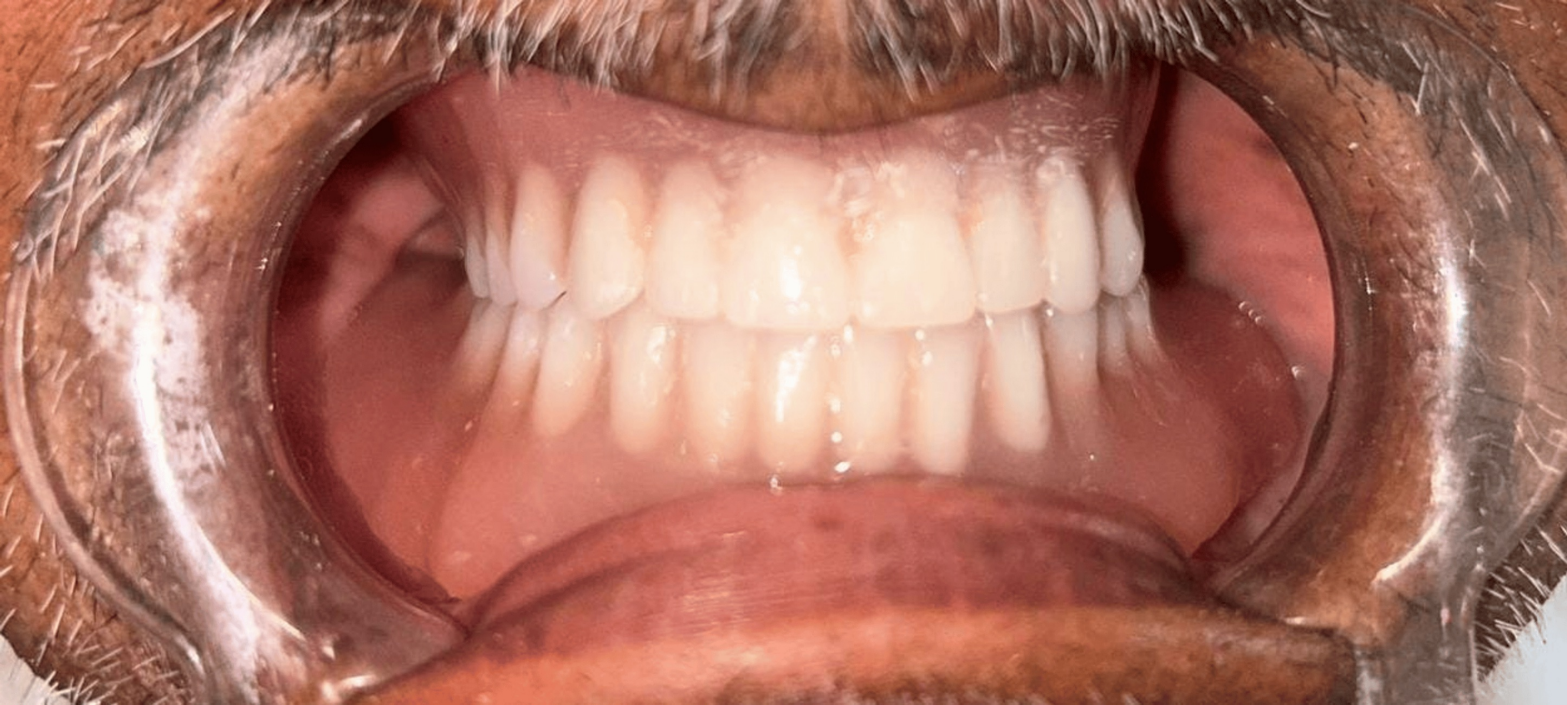
Final prosthesis was delivered to the patient

In addition, maxillary arch border molding was performed with the low-fusing impression compound, and the final impression was made with light body PVS impression material (Dentsply Reprosil Impression Material) (Figure 3d). All the steps were performed after the final impression in a conventional manner to fabricate the complete denture prosthesis. The denture was delivered to the patient (Figure 4) with the postinsertion instruction, and a follow-up appointment was scheduled for the patient within 24 hours to evaluate the denture fitting and address any discomfort or issues.

## Discussion

Ensuring the firmness and retention of dentures is a great challenge in managing flabby ridges of complete denture patients. To understand how much flabbiness affects the fit of those dentures, one has to inspect thoroughly the patient’s oral cavity [7]. A detailed case history apart from a physical examination is important in appreciating the progressiveness of these flaccid ridges as well as past attempts made on their management by prostheses. To come up with a full treatment plan subsequent to an assessment may involve tissue conditioning, modified impression techniques, and resilient denture liners, among others. It is crucial, nonetheless, that response intervention be monitored and treatment be individualized for all patients involved [8].

There are instances when surgical intervention is required to manage extremely flabby ridges, requiring collaboration between restorative dentists and oral surgeons or periodontists to cater to every aspect of a patient’s welfare [9]. For example, regular follow-up appointments and adjustments might be needed to maintain an optimum fit and function of the denture. Implant placement can also be considered as part of treatment planning in cases with flabby tissue, resulting in notable improvements in prosthesis stability and comfort. Additionally, implants provide a firm foundation that holds prosthetics firmly within the mouth, making them appropriate, especially when there is a loss of soft tissue support around dentures.

Further insight into perfecting impression techniques comes through the technique by McCord and Grant described earlier, which shows how it can be done [10]. These techniques illuminate the potential for enhanced precision and accuracy in capturing the displaced tissues, ultimately contributing to improved denture fit and comfort. A close-fitting custom tray with a window is utilized in the window technique to create the impression of a flabby ridge, as shown in the work by Watt and MacGregor [11]. Osborne detailed a method in which the "flabby" and "normal" tissues were recorded independently using two different impression trays and materials, and the results were then compared intraorally [12]. Liddelow's management of flabby tissue includes various impression techniques for a special tray, and two different impression materials (applying zinc oxide eugenol [ZOE] to the normal tissues and "plaster of Paris" to the flabby tissues) were used [13].

Studies on the window technique have suggested recording an impression and the peripheral seal, then preparing the window and recording the displaced tissues using an impression material with a low viscosity (impression plaster) [7]. Lynch and Allen suggested that a special tray with a window be made before the final impression was made and that, after the final impression, movable tissue should be recorded in a stationary position through the window [9]. The selective pressure impression technique is an additional technique wherein the area with flabby tissue is given more relief. A custom tray is then made over the spacer, and trays made of clear acrylic are applied to the area where double spacers are applied. The final impression is then made using monophase PVS material, as shown by Pai et al. [14]. In the selective perforation tray technique, in this instance, the normal tissue is recorded using ZOE, the flabby tissue is recorded using light body PVS, and the posterior palatal seal region and peripheral boundary are recorded using impression compound, etc. [15].

In this case report, Zafarullah and Hobkirk have modified Watson's window impression technique to capture the flabby tissues in their resting state more precisely. They crafted a custom tray featuring an aperture over the flabby region, commonly the anterior area, and further modified it by adding perforations over this same section of the tray. Wash impression was made using the PVS impression materials. This material covers the flabby tissue, capturing minute details and guaranteeing a precise record of the denture-bearing region because PVS has a low viscosity and does not displace the mobile tissue.

## Conclusions

For patients with complete dentures, the Zafarullah treatment provides a conservative and efficient method of treating flabby ridges. This method assures a stable and comfortable denture fit by reducing tissue displacement and emphasizing precise impression procedures. For dentists looking to enhance the results of denture treatment in patients with hypermobile ridge tissue, it is an invaluable tool. In summary, treating flabby ridges in patients wearing complete dentures is a complex procedure that calls for a customized treatment plan and a thorough understanding of the patient's oral anatomy. Dentists can assist patients in achieving the best possible denture fit, function, and comfort by addressing the unique problems caused by flabby ridges.
